# Supplementary material for: Effects of Carbohydrate and Protein Administration by Food Items on Strength Response after Training in Stable COPD
Source: Nutrients. 2022 Aug 30;14(17):3565. doi: 10.3390/nu14173565 (PMC9460301; doi:10.3390/nu14173565)
Supplement: Supplementary file 1 [file nutrients-14-03565-s001.zip › nutrients-1859487-supplementary.pdf]

Table S1. Anthropometric data

ID	sex	Age	BMI	FEV1 (%)	GOLD	mMRC	6MWT (m)	6MWT (%)	BODE	CAT
1	w	69	27.31	59	2	1	416	71	5	19
2	m	72	24.00	38	3	2	491	76	7	20
3	w	72	29.69	68	2	2	402	81	5	22
4	m	57	39.51	66	2	2	464	70	5	28
5	m	67	29.04	86	1	0	498	75	4	16
6	w	55	23.53	28	4	4	550	80	10	22
7	m	59	30.39	47	3	0	500	72	6	23
8	w	66	28.09	50	2	1	593	88	5	12
9	w	56	18.22	25	4	4	320	47	12	23
10	m	74	20.90	70	2	1	500	72	5	15
Mean		64.7	27.1	53.7			473.4	73.2		
±SD		±7.3	±5.9	±19.7			±77.8	±10.8		
Median					2	1.5			5	21
(min; max)					(1;4)	(0;4)			(4;12)	(12;28)
